# Parkinson’s disease impairs cortical sensori-motor decision-making cascades

**DOI:** 10.1093/braincomms/fcae065

**Published:** 2024-03-14

**Authors:** Alessandro Tomassini, Thomas E Cope, Jiaxiang Zhang, James B Rowe

**Affiliations:** 1 MRC Cognition and Brain Sciences Unit, University of Cambridge, Cambridge CB2 7EF, UK; 1 MRC Cognition and Brain Sciences Unit, University of Cambridge, Cambridge CB2 7EF, UK; Department of Clinical Neurosciences, University of Cambridge, Cambridge CB2 0SZ, UK; Department of Neurology, Cambridge University Hospitals NHS Trust, Cambridge CB2 0QQ, UK; Department of Computer Science, Swansea University, Swansea SA18EN, UK; 1 MRC Cognition and Brain Sciences Unit, University of Cambridge, Cambridge CB2 7EF, UK; Department of Clinical Neurosciences, University of Cambridge, Cambridge CB2 0SZ, UK; Department of Neurology, Cambridge University Hospitals NHS Trust, Cambridge CB2 0QQ, UK

**Keywords:** Parkinson’s disease, accumulator, uncertainty, visuomotor, decision-making

## Abstract

The transformation from perception to action requires a set of neuronal decisions about the nature of the percept, identification and selection of response options and execution of the appropriate motor response. The unfolding of such decisions is mediated by distributed representations of the decision variables—evidence and intentions—that are represented through oscillatory activity across the cortex. Here we combine magneto-electroencephalography and linear ballistic accumulator models of decision-making to reveal the impact of Parkinson’s disease during the selection and execution of action. We used a visuomotor task in which we independently manipulated uncertainty in sensory and action domains. A generative accumulator model was optimized to single-trial neurophysiological correlates of human behaviour, mapping the cortical oscillatory signatures of decision-making, and relating these to separate processes accumulating sensory evidence and selecting a motor action. We confirmed the role of widespread beta oscillatory activity in shaping the feed-forward cascade of evidence accumulation from resolution of sensory inputs to selection of appropriate responses. By contrasting the spatiotemporal dynamics of evidence accumulation in age-matched healthy controls and people with Parkinson’s disease, we identified disruption of the beta-mediated cascade of evidence accumulation as the hallmark of atypical decision-making in Parkinson’s disease. In frontal cortical regions, there was inefficient processing and transfer of perceptual information. Our findings emphasize the intimate connection between abnormal visuomotor function and pathological oscillatory activity in neurodegenerative disease. We propose that disruption of the oscillatory mechanisms governing fast and precise information exchanges between the sensory and motor systems contributes to behavioural changes in people with Parkinson’s disease.

## Teaser

Tomassini *et al*. report that Parkinson's disease disrupts the beta-frequency activity mediating the accumulation of evidence for decision-making, leading to inefficient processing and transfer of perceptual information to the frontal cortex, and atypical decision-making. These findings highlight the connection between changes in neural dynamics and visuomotor function in neurodegenerative disease.

## Graphical Abstract

**Graphical Abstract:**
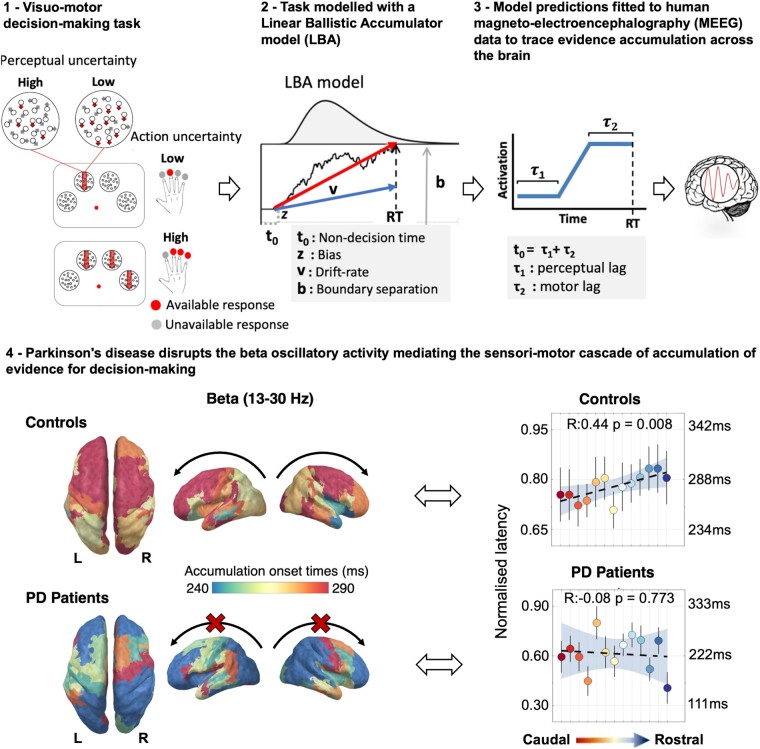

## Introduction

Parkinson’s disease is a neurodegenerative disorder characterized by the motor features of tremor, rigidity, bradykinesia and postural instability, associated with dopaminergic deficiency in the basal ganglia. However, cognitive and behavioural changes are common and early aspects of the disease, even before clinically manifest cognitive impairment and dementia. Impairments include processing speed, simple perceptual and complex behavioural decisions,^1,2^ attention and selection of actions^3^ and inhibitory control,^4^ which together underlie a wide range of executive function deficits.^5^ The cognitive processes affected by Parkinson’s disease are not necessarily restricted to those mediated by neurons in the basal ganglia foci of early pathology, but include widely distributed cortical networks.

The cognitive processes underlying the deficits in Parkinson’s disease can be conceived as a set of neuronal decisions, linking perception of environmental cues to the selection and execution of appropriate actions. Even apparently ‘simple’ decisions unfold via sequential overlapping processes,^6,7^ establishing a cascade in which the accumulation of evidence for perceptual decisions informs the accumulation of evidence (or ‘intentions’) for selection of a motor response.^8-12^ This concept gains further relevance in light of studies suggesting that premature response choices in Parkinson’s disease patients can be mediated by early visuomotor activation of the motor cortex^13^ that can “leak” into the motor periphery.^14^ The spatiotemporal overlap plays a crucial role in balancing the trade-off between robust but slow serial processes and fast but error-prone parallel processes, ultimately influencing the speed and accuracy of responses. Departures from optimal spatiotemporal overlap may bias the system towards slower or more inaccurate responses.^13-15^

Spatially distributed processing exploits neural oscillations for effective communication between regions.^16^ Large-scale oscillatory activity at beta (∼13–30 Hz) and more localized activity at gamma (∼30–90 Hz) frequency ranges have been particularly associated with feedback and feed-forward information transfer, respectively.^17-19^ Changes in spectral power and disruption of functional brain network organization occur in Parkinson’s disease. For example, exaggerated oscillations at beta frequency are a signature of pathology in the basal ganglia and frontoparietal network in Parkinson’s disease patients^20,21^ (for a review, see Schnitzler and Gross^22^).

Dopaminergic deficiency in Parkinson’s disease may contribute to the generation of aberrant oscillatory activity in both the beta and gamma ranges.^23^ A feature of pathological oscillations in Parkinson’s disease is the reduced power modulation elicited by changes in environmental and cognitive demands. Such inflexibility may contribute to cognitive changes in Parkinson’s disease.^24^ This mirrors normative accounts of decision-making. For example, sequential sampling models of behaviour in which deficits are associated with inflexibility of the latent cognitive decision processes estimated from patients’ performance on experimental tasks.^2,25,26^ Distributed frontoparietal networks are involved in the selection and accumulation of evidence to reach decisions in non-human primates^11^ and humans.^27,28^ Abnormalities in these circuits arise early in Parkinson’s disease. Their functional significance is revealed by integrating neurophysiology (electroencephalography [EEG] and/or magnetoencephalography [MEG]) with models of behaviour in which latent cognitive processes cannot be observed directly.^29^

Here we adopted linear ballistic accumulator (LBA) models to identify latent components of cognitive processes^30^. The LBA posits separate accumulator processes, accruing evidence for alternatives in a race to reach the decision boundary. The fastest accumulator resolves the decision. In addition to time spent in evidence accumulation, there are ‘non-decision processes’ before and after. The non-decision time includes sensory encoding and motor execution processes. These are not typically distinguished in behavioural modelling,^31-33^ but it is possible to do so.^29^ Decomposing the non-decision time enables one to examine the spatial distribution of the latency to accumulation.^34^ This in turn enables the separation of the effect of spatially inhomogeneous disease on perceptual encoding versus noise of accumulators.^2,25,34,35^ The transformation of perceptual decisions into action selection across the dorsal stream may be especially susceptible to the dopaminergic effect of Parkinson’s disease.^11,27,36^ We predict that the effect of Parkinson’s disease on the spatiotemporal cascade of evidence accumulation would not only be seen within frontostriatal circuits but also posterior cortex in receipt of frequency-bound top-down signalling from the frontal cortex.

**Figure 1:**
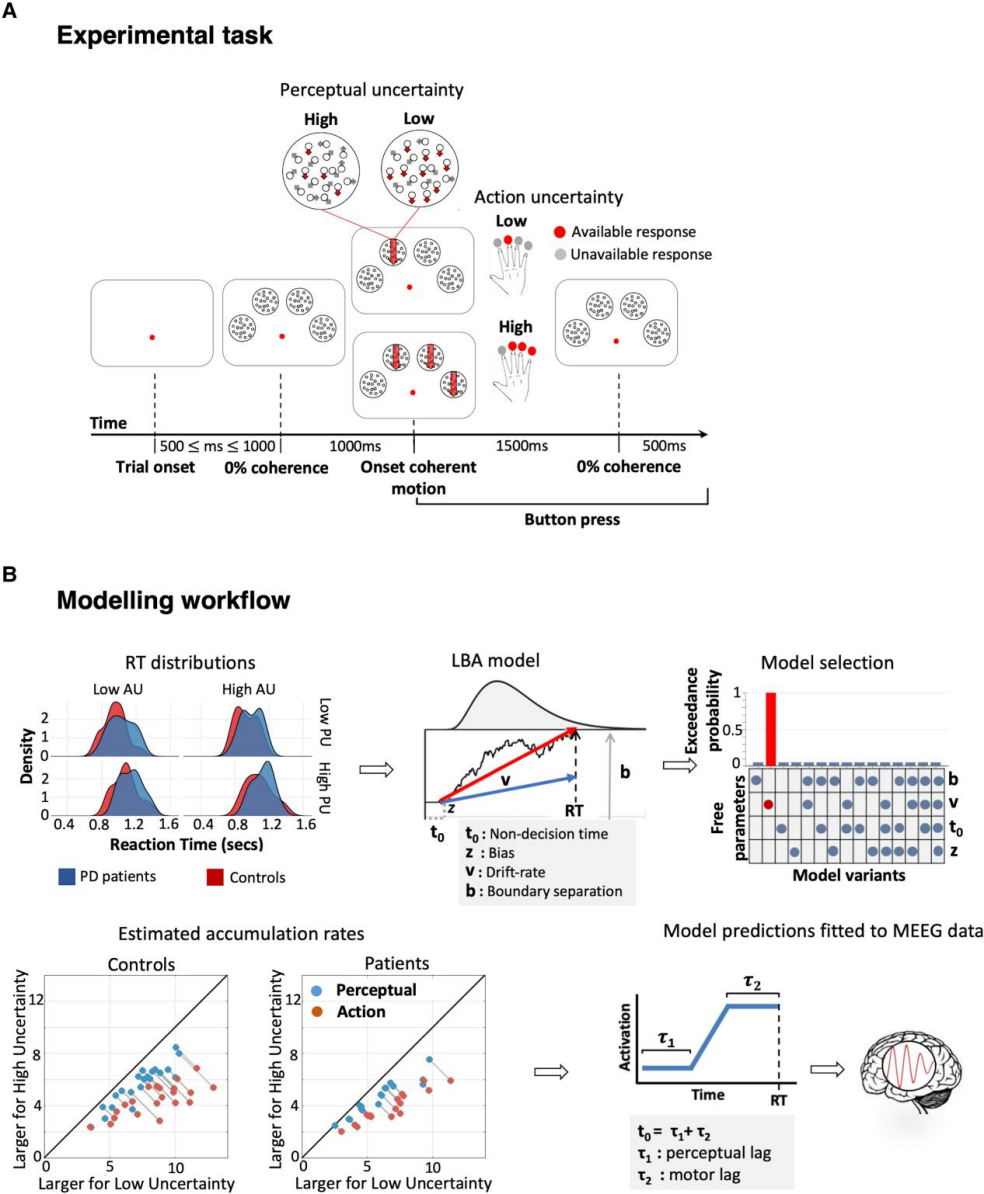
**Experimental task and modelling procedure.** (**A**) Participants pressed the button corresponding to the coherent stimulus (here indicated by downward arrows). When there was more than one coherent stimulus, they selected one response and pressed the corresponding button. Perceptual uncertainty (PU) was manipulated by changing the coherence of dot motion (i.e. by changing the motion strength), whereas action uncertainty (AU) was manipulated by changing the number of available options (i.e. the number of coherent stimuli to choose from). Perceptual and action uncertainty varied across trials in a 2 by 2 factorial design. (**B**) Reaction times from each participant (upper left panel) were modelled using a linear ballistic accumulator (LBA) model (upper *middle* panel). Noisy evidence accumulates over time at a rate *v* up to a decision bound *b*. The fastest accumulator (thick red arrow) determines the choice. The bias *z* accounts for individual preferences towards a given motor response. Non-decision time linked to sensorial and motor processes (*t*_0_) sums to the evidence accumulation time to produce reaction times. The model best accounting for the behavioural data was selected with Bayesian model comparison (upper right panel): changes in the sole accumulation rate best accounted for the behavioural data. For both controls and patients, the model predicted faster accumulation of decision-evidence when uncertainty is low for both action and perceptual uncertainty (lower left panel; grey lines connect data points from each participant; the plots show accumulation rates estimated for low uncertainty in the *x*-axis and high uncertainty in the *y*-axis; points below the diagonal indicate larger accumulation rates for low uncertainty and vice versa). Neural activity simulated with the winning model was fitted to the power envelope of the MEEG signal in a trial-by-trial fashion to identify the latencies of accumulators of decision-evidence. Non-decision time (*t*_0_) was decomposed into pre-accumulation (*τ*_1_) and post-accumulation (*τ*_2_) time reflecting perceptual and motor processes, respectively (lower right panel).

To test this prediction, we used electro-magnetoencephalography (MEEG) in people with Parkinson’s disease, without dementia, on their usual medication. In the visuomotor decision task, noisy visual stimuli indicate one or more permitted manual response options (Fig. 1A). We independently manipulated perceptual uncertainty and the number of permitted actions.^19^ This separated variance due to the neural signatures of perceptual decisions (e.g. ‘which options are available?’) from action decisions (e.g. ‘which option do I choose?’), without *a priori* spatial bias to ‘sensory’ or ‘motor’ cortical regions. The spatiotemporal pattern of decision onsets was identified by optimizing the division of the non-decision time to before versus after the accumulation period, according to trial-specific profiling of the induced power.^19^

We predicted that people with Parkinson’s disease have shorter sensorial processing latency relative to controls^37,38^ and differential spatial gradients in the onset of frequency-specific accumulation across the dorsal stream. Whereas controls modulate the latent cognitive processes in response to the different levels of uncertainty (indexed by the LBA modelling), we predicted relative insensitivity to task modulation of uncertainty in people with Parkinson’s disease, in view of the influence of dopaminergic regulation of beta and gamma mediators of cognitive control.^17-19,23,24^

## Materials and methods

### Participants

Forty-four participants were recruited, comprising 21 patients with idiopathic Parkinson’s disease (UK Brain Bank clinical diagnostic criteria) and 23 age-matched healthy controls. Four patients and two controls were excluded due to poor-compliance in-scanner with the task, or technical issues during the scanning, resulting in 17 analysed patient datasets and 21 analysed control datasets. Inclusion criteria were age 50–80 years, right-handed and no previous history of neurological or psychiatric illness apart from Parkinson’s disease. The revised Addenbrooke’s Cognitive Assessment Scale (ACE-R), including the mini-mental state examination (MMSE), was administered to all participants. No patients had clinical presentations of dementia, and there was no difference in ACE-R scores between groups (Table 1). Patients were also assessed on the Unified Parkinson’s disease rating scale.^39^ Clinical assessments and experimental tasks were performed on usual dopaminergic medication, and dopaminergic dose equivalents were calculated.^40^ Experimental protocols including written informed consent conformed to the guidelines of the Declaration of Helsinki and were approved by the Cambridge Research Ethics Committee (CRE code: 07/H0307/64).

**Table 1:** Demographic and clinical features of participants

		Controls	Patients	Group difference
Male/female		13/8	10/7	n.s.
Age		67 ± 6.9 (51–75)	63 ± 7.5 (50–77)	n.s.
MMSE (30)		29.9 ± 0.3 (29–30)	29.8 ± 0.2 (28–30)	n.s.
ACE-R (100)		96.4 ± 3.5 (85–100)	97 ± 2.6 (83–100)	n.s.
	Attention (18)	17.9 ± 0.2 (17–18)	17.9 ± 0.2 (17–18)	n.s.
	Memory (26)	24.2 ± 2.1 (20–26)	24.5 ± 2.1 (15–26)	n.s.
	Verbal fluency (14)	12.6 ± 1.6 (7–14)	12.9 ± 1.6 (10–14)	n.s.
	Language (26)	25.9 ± 0.3 (25–26)	25.9 ± 0.3 (25–26)	n.s.
	Visual spatial (16)	15.7 ± 0.6 (14–16)	15.8 ± 0.4 (14–16)	n.s.
UPDRS	Total	n.a.	39.6 ± 15.3 (18–61)	
	Part III motor sub-scale	n.a.	26.2 ± 12.6 (13–54)	
	Hoehn & Yahr stage	n.a.	1.64 ± 0.5 (1–2)	
Symptomatic laterality Left/right		n.a.	9/8	
Levodopa equivalent daily dose (LEDD)		n.a.	266 ± 127.2 (0–750)	
Years from symptom onset		n.a.	5.9 ± 4.1 (1–15)	

Values shown are group means and their standard deviations (range in parentheses). MMSE, 30-point mini-mental state examination; ACE-R, 100-point Addenbrooke’s cognitive exam revised, divided into five subscales with maximum points in parentheses; UPDRS, Unified Parkinson’s disease rating scale. Group differences were tested using Wilcoxon rank-sum test.

### Stimuli

Stimuli were presented using Matlab (MathWorks, Natick, MA, USA) and Psychtoolbox in a quiet and dimly lit room. For training, stimuli were displayed on a cathode-ray tube (CRT-type) monitor at 60 cm distance from the observer. For the scan session, stimuli were projected on a screen at 130 cm distance (60 Hz refresh rate) with equivalent pixel resolution of 0.03°. On each trial, four random dot kinematograms were displayed within four circular apertures (4° diameter) positioned along a semi-circular arc (3.4° eccentricity) on a black background. Two hundred dots were displayed during each frame and spatially displaced to introduce apparent downward motion (6°/s velocity). Motion coherence was manipulated by allowing only a certain proportion of dots to move downward on each frame whilst the rest were randomly reallocated. Motion coherence level was varied across trials. The 1.5 s long coherent motion interval was preceded and followed by intervals of zero-coherence levels lasting 1 s and 0.5 s, respectively. This was to avoid large stimulus sensory-evoked potentials elicited by abrupt stimulus onset/offset, which might mask decision processes.

### Psychophysical assessment of motion sensitivity

A practice session adopting 100% coherent stimuli allowed participants to familiarize themselves with the task. The practice session ended once participants reached 90% accuracy across all trial types. In the following psychophysical session, motion coherence was varied between trials to estimate individual motion thresholds. Eight logarithmically spaced motion coherence levels (0 0.5 0.10. . .0.9) were used (32 trials per level). Each training session comprised 16 blocks of 32 trials. Feedback was provided for correctness of responses as well as for too early or too late responses (100 ms and 2.5 s from motion coherence onset, respectively).

The psychophysical task was combined with a control match-to-sample task where occasionally (*P* = 0.2) after a correct choice, participants had to compare the location of a set of grey discs with the location of the previously displayed coherent stimuli. This was to ensure that participants perceived all the available options (i.e. coherent stimuli) before committing to a decision. To report a match, participants had to press any button and withhold a response otherwise. A trial was considered as correct only when both choice and matching were correct. Trials with un-matching responses were discarded and repeated within the session.

To tailor low and high levels of perceptual uncertainty to individual motion sensitivity across number of coherent stimuli, the motion-coherence dependent accuracy of each trial type (e.g. either 1 or 3 coherent stimuli) was fitted using a maximum likelihood method, with the Log-Quick function


(1) $$F_{\log}=1-2^{-10^{\beta (x-\alpha )}}$$

where *α* is the threshold, *β* is the slope and *x* is the coherence level. To obtain the correct proportion for each trial type, $F_{\log}$ was scaled by


(2) $$P=\gamma +(1-\gamma -\lambda )F_{\log}$$

where *γ* is the guess rate, and *λ* is the lapse rate controlling the lower and upper asymptote of the psychometric function, respectively.

Individual low and high perceptual uncertainty levels for each trial type were estimated as the 75th and 90th percentiles of the psychometric function.^19^

### Task and procedures

Participants performed a task in which they had to report a downward coherent stimulus by pressing the corresponding button.^19^ The number of available coherent stimuli defined two trial types: low action uncertainty trials, where a single coherent stimulus commanded which button to press, and high action uncertainty trials, where three coherent stimuli required the participants to press any one of the three corresponding buttons (making a ‘fresh choice, regardless of what you have done in previous trials’). Equal emphasis was placed on the speed and accuracy of the responses, and guessing was discouraged. Participants were instructed to fixate on a central red mark throughout the trial. Each trial started with the presentation of the fixation mark and stimuli onset ensued after a variable interval comprised between 0.5 s and 1 s. The imaging session was preceded by one training psychophysical session scheduled on a separate day (maximum 4 days between sessions). In the scan sessions, coherence levels were fixed to the individual motion thresholds corresponding to high and low levels of perceptual uncertainty, the match-to-sample task was removed, and no feedback was provided except for too early or too late responses. Levels of perceptual and action uncertainty were randomly interspersed across trials. Each session consisted of 10 blocks (total 640 trials per participant) separated by a short rest (total duration of the session ∼60 minutes).

### Voxel-based morphometry

Following MEEG, participants underwent structural MR imaging using a 3T Siemens Tim Trio scanner with a 32-channel phased-array head coil. A T_1_-weighted magnetization-prepared rapid gradient-echo image was acquired with repetition time (TR) = 2250 ms, echo time (TE) = 3.02 ms, matrix = 192 × 192, in-plane resolution of 1.25 × 1.25 mm, 144 slices of 1.25 mm thickness, inversion time = 900 ms and flip angle = 9°. Images were first aligned to a canonical average image in MNI space, before segmentation and calculation of total intracranial volume (TIV). After segmentation, a study-specific DARTEL template was created from the patient scans and the seventeen closest age-matched controls. The remaining controls were then warped to this template. The templates were aligned to the SPM standard space and the transformation applied to all individual modulated grey-matter segments together with an 8 mm FWHM Gaussian smoothing kernel.

### MEG and EEG data acquisition and processing

An Elekta Neuromag Vectorview System (Helsinki, Finland) simultaneously acquired magnetic fields from 102 magnetometers and 204 paired planar gradiometers, and electrical potential from 70 EEG electrodes (70 Ag-AgCl scalp electrodes in an Easycap—GmbH, Herrsching, Germany—extended 10–10% system). Additional electrodes provided a nasal reference, a forehead ground, paired horizontal and vertical electro-oculography, electrocardiography and right arm electromyography. All data were recorded and digitized continuously at a sample rate of 1 kHz and high-pass filtered above 0.01 Hz.

Before scanning, head shape, the locations of five evenly distributed head position indicator coils, EEG electrodes location and the position of three anatomical fiducial points (nasion and left and right pre-auricular) were recorded using a 3D digitizer (Fastrak Polhemus Inc., Colchester, VA). The initial impedance of all EEG electrodes was optimized to below 10 kΩ, and if this could not be achieved in a particular channel, or if it appeared noisy to visual inspection, it was excluded from further analysis. The 3D position of the head position indicators relative to the MEG sensors was monitored throughout the scan.

These data were used by Neuromag Maxfilter 2.2 software,^41^ to perform environmental noise suppression, motion compensation and Signal Source Separation. Subsequent analyses were performed using in-house Matlab (MathWorks) code, SPM12 (www.fil.ion.ucl.ac.uk/spm) and EEGLab^42^ (Swartz Center for Computational Neuroscience, University of California San Diego). Artefact rejection was performed through separate independent component analysis decomposition for the three sensor types. For EEG data, components temporally and spatially correlated to eye movements, blinks and cardiac activity were automatically identified with EEGLab’s toolbox ADJUST.^43^ For MEG data, components were automatically identified that were both significantly temporally correlated with electro-oculography and electrocardiography data, and spatially correlated with separately acquired topographies for ocular and cardiac artefacts. A further independent component analysis identified that MEG and EEG components significantly temporally correlated with tremor-related surface electromyographic activity (2–8 Hz) recorded in Parkinson’s disease patients. Artefactual components were finally projected out of the dataset with a translation matrix.

The continuous artefact-corrected data were low-pass filtered (cut-off = 100 Hz, Butterworth, fourth order), notch filtered between 48 and 52 Hz to remove main power supply artefacts, down-sampled to 250 Hz and epoched from −1500–2500 ms relative to motion coherence onset. EEG data were referenced to the average over electrodes. MEG and EEG data were combined before inversion into source space^44^ using the Minimum Norm algorithm as implemented by SPM12. Notably, combined MEG and EEG allows a better localization of neural sources than each technique on its own.^44^ The forward model was estimated from each participant’s anatomical T_1_-weighted MRI image. All conditions were included in the inversion to ensure an unbiased linear mapping. The source images were spatially smoothed using an 8 mm FWHM Gaussian kernel.

### Modelling of perceptual and action decisions

To decompose behavioural performance into latent variables underpinning decisions, we fitted LBA models to each participant’s reaction time data. The LBA model belongs to the broad class of accumulation-to-threshold models of decision-making but is more tractable than drift-diffusion models for n-way decisions while still remaining physiologically informative.^45^ We followed the same logic of previous work^19^ and opted for a ‘unitary’ model where both perceptual and action uncertainty concur in determining a participant’s performance in a given trial. The unitary model accommodated the empirical data under the assumption of a constant non-decision time across experimental conditions, which provides theoretical evidence against the need of an extra stage of processing.^46^ Accordingly, each accumulator linearly integrates the decision-evidence (or the intention) over time in favour of one action, and the decision is made when the accumulated activity reaches a threshold (see Fig. 1B). In our task, possible actions correspond to a button press from one of four fingers, each modelled by independent accumulators. When three valid actions are available, three accumulators are engaged with activation starting at levels independently drawn from a uniform distribution, and increasing linearly over time with an accumulation rate *v* drawn from an independent normal distribution. A response is triggered once one accumulator wins the ‘race’ and reaches a decision bound *b*. When only one action is available, only the accumulator corresponding to the available action is engaged. Predicted reaction time is given by the duration of the accumulation process for the winning accumulator, plus a constant non-decision time *t*_0_ representing the latency associated with stimulus encoding and motor response initiation.

The LBA model was fitted to the behavioural data using a bounded version of MATLAB’s fminsearch function. We employed the Maximum Likelihood Estimation (MLE) procedure through a custom MATLAB module. To enhance numerical convergence, we adopted multi-start optimization. This method was uniformly applied to every model variant and participant dataset, with 25 optimization runs for each. Each optimization run was preceded by 100 random searches to determine the best starting parameter values. The reliability of our findings was verified by repeated optimization sessions converging on similar solutions, allowing minor numerical variations.

### Dimensionality reduction

To improve computational efficiency and reduce multiple comparisons, we reduced the dimensionality of the MEEG data by parcellating the cortical surface into a set of 96 regions of interest (ROIs) defined using the Harvard-Oxford cortical atlas (FSL, FMRIB, Oxford). The dynamic of each ROI was represented by a single time-course, obtained by extracting the principal component from the vertices lying within the given ROI.^19^ The reconstructed sources within each ROI were first bandpass-filtered in either beta (13–30 Hz) or gamma (31–90 Hz) frequency bands. The coefficients of the principal component accounting for the majority of the variance of the vertices within each ROI were then taken as an appropriate representation of source activity for that region. Next, to estimate the power oscillations on a single-trial basis, we extracted frequency-specific signal envelope modulations using a Hilbert transform of the source data from each reconstructed ROI (epochs from 500 ms before to 1500 ms after coherence onset). The Hilbert’s envelope is a convenient measure of how the power of the signal varies over time in the frequency range of interest, and thus particularly suited to capture relatively slow fluctuations associated to the instantaneous accumulation of evidence/intentions. The power estimates of individual participants were down-sampled to 100 Hz and normalized by their baseline (from 400–100 ms before coherence onset).

### Statistical analysis

For all the frequentist statistical analyses, the threshold of statistical significance was set to α = 0.05, corrected for multiple comparisons where appropriate.

#### Analysis of demographic and clinical characteristics

We tested whether the groups in the study were matched based on demographic characteristics, namely gender and age, and cognitive performance, measured by the ACE-R cognitive test. For the comparison of gender, we used the Fisher’s exact test. We also calculated the odds ratio to quantify the strength of the association of gender with the two groups under consideration. An odds ratio of ∼1 indicates no significant association between the two. To compare the ages and ACE-R test score of the participants between groups, we used the non-parametric Wilcoxon rank-sum test. For all the tests, the *P*-values were calculated two-tailed.

#### Assessing anatomical changes in Parkinson’s disease

**Figure 2:**
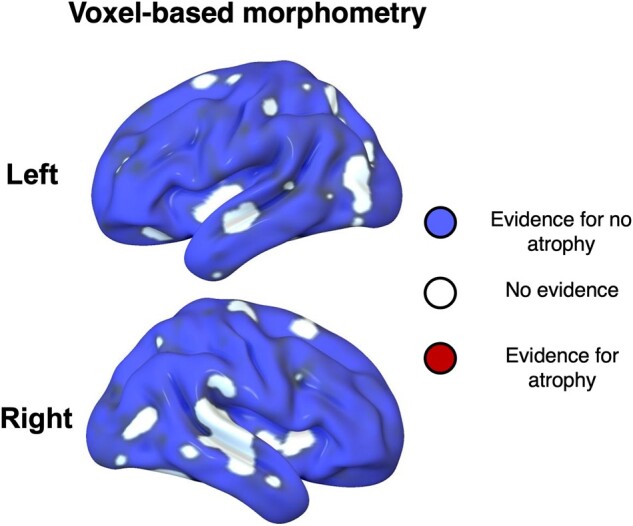
**Bayesian voxel-based morphometry in the patient group.** Areas in blue had substantial evidence for normal cortical volume in Parkinson’s disease patients (posterior probability *P* > 0.95 for the null voxel-wise threshold > 0.7, cluster volume > 1 cm^3^). White areas had no substantial evidence for or against atrophy. Red areas (not present) would indicate substantial evidence for atrophy.

The resulting images from the VBM processing were entered into a full factorial general linear model with a single ‘group’ factor of two levels, and age and TIV as covariates of no interest. This model was estimated in two steps. Firstly, a classical (frequentist) estimation was performed and a T-contrast constructed to compare groups. Cluster-level inference was performed by setting the voxel-wise cluster-forming ‘height’ threshold *P* < 0.001 uncorrected (height threshold *t* = 3.39, extent threshold *k* = 0). Clusters were defined as atrophic if they were significant at the cluster family-wise error (FWE)-corrected *P* < 0.05 level. Secondly, a Bayesian estimation was performed on the same model, and a Bayesian F-contrast between patients and controls specified. The resulting Bayesian map was subjected to hypothesis testing for the null (i.e. lack of atrophy) in SPM12, resulting in a map of the posterior probability of the null at each voxel. For visualization in Fig. 2, this map was thresholded Bayesian posterior probabilities of the null above voxel-threshold effect 0.7 and cluster volumes of >1 cm^3^ (default settings in SPM).

#### Behavioural analysis

To assess were significant differences in individual motion coherence thresholds, RT and accuracy between the two groups, we applied mixed-design repeated-measures ANOVA with Group (PD, controls) as between factor and PU, AU (both with levels Low, High) as within factors.

For all ANOVA tests implemented in this study, generalized eta-squared ( ${\hat{\eta }}_{G}^{2}$) was estimated to give an assessment of the effect size of our findings. Moreover, we also calculated Bayes Factors (BF) to provide a quantitative measure of the evidence for or against the null hypothesis.

#### Model comparison

To identify the combinations of free parameters that best accounted for the observed behavioural data, we repeatedly fitted the LBA model with 15 unique combinations of free parameters (i.e. all possible combinations without repetition) allowed to vary across conditions. The best-fitting parameters for each model variant were used to calculate the Bayesian Information Criterion (BIC), as a measure of goodness of fit that penalizes extra free parameters in favour of simpler models.

To adjudicate the best-fitting model variant, we adopted Bayesian random-effect analyses on the BIC values obtained by the fitting procedure.^47,48^ This approach permits one to quantify the evidence for the explanatory power (i.e. frequencies) of each model across participants and groups.^49^ In a first step, we assessed whether model frequencies were the same ( H _=_) or differed ( $H_{\neq }$) between the two groups. Under ( H _=_), data are assumed be generated by the same model, therefore a standard random-effect analysis on the datasets pooled across groups yields the (log) evidence $p(\mathrm{Data}\;|\;H_{=})$. Under $H_{\neq }$, group-specific datasets are assumed to be generated by different models and thus the (log) evidence $p(\mathrm{Data}\;|\;H_{\neq })$ is defined as the product of group-specific evidences $p(\mathrm{Controls}\;|\;H_{=})+p(\mathrm{Patients}\;|\;H_{=})$ obtained by a separate random-effect analysis for each group. The posterior probability that the same model is valid for both groups is then given by


(3) $$P(H_{=}\;|\;\mathrm{Data})=\frac{1}{1+\exp(\;p(\mathrm{Data}|H_{\neq })-p(\mathrm{Data}|H_{=}))}.$$

The results from the between-group model comparison confirmed that the data were generated by the same model across groups (see ‘Results’). Therefore, in a second step, we performed a random-effect analysis on the pooled dataset to identify the model variant prevailing in the population. The prevalence of the model was quantified as ‘exceedance probability’, defined as the probability that any given model is more likely than all other models.^47^ Predictions of decision-related activity were generated from the winning LBA model to locate neural signatures of decisions-evidence accumulation in single-trial analyses of MEEG data.^19,50^

#### Assessing the effects of uncertainty on power

To elucidate the impact of our task manipulations on power amplitude, we first averaged envelopes across trials and ROIs belonging to the dorsal path, and compared activity between low and high levels of action and perceptual uncertainty within the time window 0.1–1 s from coherence onset, separately within each group. Significance was estimated by cluster-corrected random permutation tests (10 000 iterations, two-tailed).

Second, to explore possible moderation effects of disease on modulation of power (median over samples), we fitted a linear mixed effects model (estimated using Maximum Likelihood) where perceptual uncertainty (PU: low, high), action uncertainty (AU: low, high) and Group (control, Parkinson’s disease) were included as fixed effects. To account for individual variations in power, as well as for variation in power between brain regions, subjects and ROIs were specified as nested random effects for the model intercept ( $\beta_{0,i|\mathrm{ROI}}$). Two interaction terms were added to test for moderation effects of Group on PU and AU separately.


(4) $$\begin{array}{rl} \mathrm{Powe}r_{i}\;= & \;\beta_{0,i|\mathrm{ROI}}+\beta_{1}*\;\mathrm{PU}+\beta_{2}\;*\;\mathrm{AU}+\beta_{3}\;*\;\mathrm{Group}\\ & +\beta_{4}\;*\;\mathrm{PU}\;*\;\mathrm{Group}+\beta_{5}\;*\;\mathrm{AU}\;*\;\mathrm{Group}+\epsilon_{i|\mathrm{ROI}}. \end{array}$$

#### Single-trial analysis

To identify the spatiotemporal profile of decision-related accumulation over the brain, we estimated the maximum lagged absolute Spearman correlation between the model predicted activity and the signal envelope in a trial-by-trial fashion.^19^ Spearman correlation was chosen for its robustness to departures from normality in the power data. The lagged correlation was used to optimally split the non-decision time before and after the accumulation period to determine the time delay between the neural signal and the model predictions. The time before accumulation provides a measure of the temporal separation between sensory encoding and onset of evidence accumulation.

We estimated the largest absolute lagged correlation value for each ROI and individuals by comparing concatenated epochs and model predictions. This choice permits the measurement of accumulation lags specific to each ROI, under the assumption that they differ across brain regions for each participant. The significance of the Fisher-transformed maximum lagged correlations for each ROI was then quantified (*Z*-score) using a non-parametric one-sample sign-test that is robust to violations of distribution symmetry. To provide a conservative estimate of significant correlations between model prediction and neural activity, we repeated the above procedure 10 000 times, each iteration using a different phase-randomized version of the original MEEG signal, to obtain a distribution of correlations under chance (null distribution). Two-tailed statistical significance was assessed by computing the proportion of absolute values from the null distribution exceeding the correlation between model predictions and the original MEEG signal. The resulting *P*-values were corrected for multiple comparisons [using FDR] across ROIs and frequency bands.

## Results

### Participant demographics

Demographic and clinical characteristics of the participants are shown in Table 1. The groups were matched for gender (Fisher’s exact test, *P*∼1.00, odds ratio = 0.88), for age (Wilcoxon rank-sum test W = 120.50, *P* = 0.13, two-tailed), and performed similarly on cognitive tests (ACE-R Wilcoxon rank-sum test W = 161.50, *P* = 0.79, two-tailed).

### Anatomical changes in Parkinson’s disease

To confirm the integrity of the network of sensory, motor and association cortices upon which our experiment relies, we used voxel-based morphometry to compare grey matter volume across the groups (Fig. 2; see Table 1 for participant characteristics). We adopted classical whole brain statistical parametric mapping *t*-test and Bayesian null tests to assess the presence or absence of structural differences between groups. Consistent with the early stage of the disease and lack of cognitive impairment, there was evidence for lack of atrophy in most of the cortex, and no significant evidence for atrophy anywhere in cortex.

### Effects of uncertainty on behavioural performance

**Figure 3:**
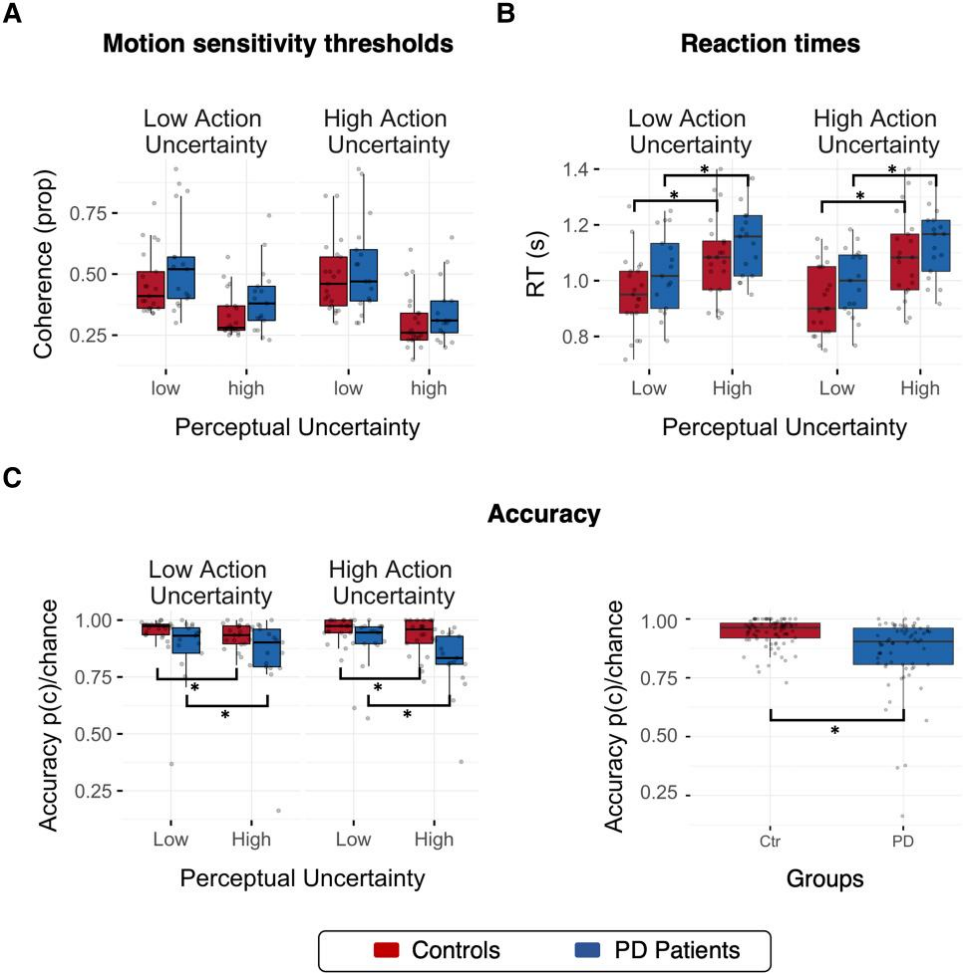
**Behavioural results.** (**A**) Motion coherence thresholds estimated from the training session did not differ between control (red) and Parkinson’s disease (PD, blue) groups (*F*(1,36) = 1.59, *P* = 0.216, ${\hat{\eta }}_{G}^{2}=0.037$, BF = 0.92). (**B**) Reaction times varied with levels of perceptual uncertainty with faster responses under low than high uncertainty (*F*(1,36) = 130.25, *P* < 0.001, ${\hat{\eta }}_{G}^{2}=0.197$, BF > 100). (**C**) Left panel: for both groups, accuracy decreased with high perceptual uncertainty (*F*(1,36) = 18.01, *P* < 0.001, ${\hat{\eta }}_{G}^{2}$ = 0.035, BF > 100); p(c) = proportion correct responses, chance = probability of correct response by guessing, accuracy = p(c)/chance. Right panel: Parkinson’s disease patients were overall less accurate than controls (*F*(1,36) = 6.77, *P* = 0.013, ${\hat{\eta }}_{G}^{2}$ = 0.130, BF = 4.68). Statistical tests performed using a mixed-design repeated-measures ANOVA with groups as between factor and uncertainty manipulations as within factor. Significance levels: *P < 0.05, ***P < 0.001.

Individual motion coherence thresholds estimated from the training session (Fig. 3) did not significantly differ between the two groups (*F*(1,36) = 1.59, *P* = 0.216, ${\hat{\eta }}_{G}^{2}=0.037$, BF = 0.92). Trials from the experimental session where responses were shorter than 100 ms or longer than 2100 ms were omitted from the behavioural and modelling analyses (controls: 15.34%, Parkinson’s disease: 20.42%). Analysis of the removed trials showed that patients did not significantly differ from controls in the number of premature responses (i.e. RT < 100 ms; Wilcoxon test: W = 397, *P* = 0.35, two-tailed) or omitted responses (W = 345, *P* = 0.06, two-tailed).

For the remaining trials, repeated-measures ANOVA showed that reaction times across groups were significantly faster for the low perceptual uncertainty condition compared with the high perceptual uncertainty condition (*F*(1,36) = 130.25, *P* < 0.001, ${\hat{\eta }}_{G}^{2}=0.197$, BF > 100) confirming the efficacy of the estimated motion coherence thresholds. We confirmed the expected lack of a significant difference between action uncertainty levels in a n-way race^51^ (*F*(1,36) = 1.99, *P* = 0.167, ${\hat{\eta }}_{G}^{2}=0.005$, BF = 0.648), and lack of interaction between action and perceptual uncertainty. There was no significant difference in reaction times across groups (*F*(1,36) = 2.46, *P* < 0.126, ${\hat{\eta }}_{G}^{2}=0.053$, BF = 1.08). Overall, Parkinson’s disease patients’ choices were less accurate than healthy controls (accuracy *F*(1,36) = 6.77, *P* = 0.013, ${\hat{\eta }}_{G}^{2}=0.130$, BF = 4.68); For both groups, accuracy decreased with high perceptual uncertainty (*F*(1,36) = 18.01, *P* < 0.001, ${\hat{\eta }}_{G}^{2}=0.035$, BF > 100).

### Evidence accumulation in Parkinson’s disease has reduced reactivity to action uncertainty

Changes in the accumulation rate alone (model two, exceedance probability = 1; Fig. 1B) best accounted for the effects of uncertainty on performance in both groups ($P(H_{=}|\mathrm{Data})$ = 0.997, BF > 100). The goodness of fit of model two (henceforth, the LBA model) was confirmed by posterior predictive checks and parameter recovery.

A mixed-design repeated-measures ANOVA on the LBA model parameters revealed slower evidence accumulation under high uncertainty levels for both action (accumulation rate: *F*(1,36) = 190.97, MSE=2.33, P<0.001, ${\hat{\eta }}_{G}^{2}=0.453$, BF > 100) and perceptual (accumulation rate: *F*(1,36) = 109.22, MSE=0.77, P<0.001, ${\hat{\eta }}_{G}^{2}=0.136$, BF > 100) manipulations. Overall, evidence accumulation was slower in the Parkinson’s disease group (*F*(1,36) = 4.69, MSE=11.41, P=0.037, ${\hat{\eta }}_{G}^{2}=0.091$, BF = 2.43), with a significant interaction between group and action uncertainty (accumulation rate: *F*(1,36) = 5.65, MSE=2.33, P=0.023, ${\hat{\eta }}_{G}^{2}=0.024$, BF = 26.95): controls showed larger changes in accumulation rates than patients in response to action uncertainty. *Post hoc* comparisons using Bonferroni-corrected *t*-tests showed that the interaction was caused by accumulation rates significantly slower in the Parkinson’s disease group compared to controls under low action uncertainty (Low action uncertainty: ΔM=1.785, t(50.10)=2.952, P=0.0048; High action uncertainty: ΔM=0.602, t(50.10)=0.995, P=0.3245).

Taken together, our results show that, regardless of group, the experimental manipulation of perceptual and action uncertainty modulated accumulation rates in line with previous reports.^2,19,50^ However, compared to healthy controls, the Parkinson’s disease group was characterized by a reduced reactivity of accumulation rates to changing uncertainty levels in the action domain. Specifically, the intention to move was accumulated significantly slower than controls under low levels of action uncertainty (i.e. when a single specific action was to be taken).

### Beta power desynchronization in Parkinson’s disease has reduced reactivity to action uncertainty

**Figure 4:**
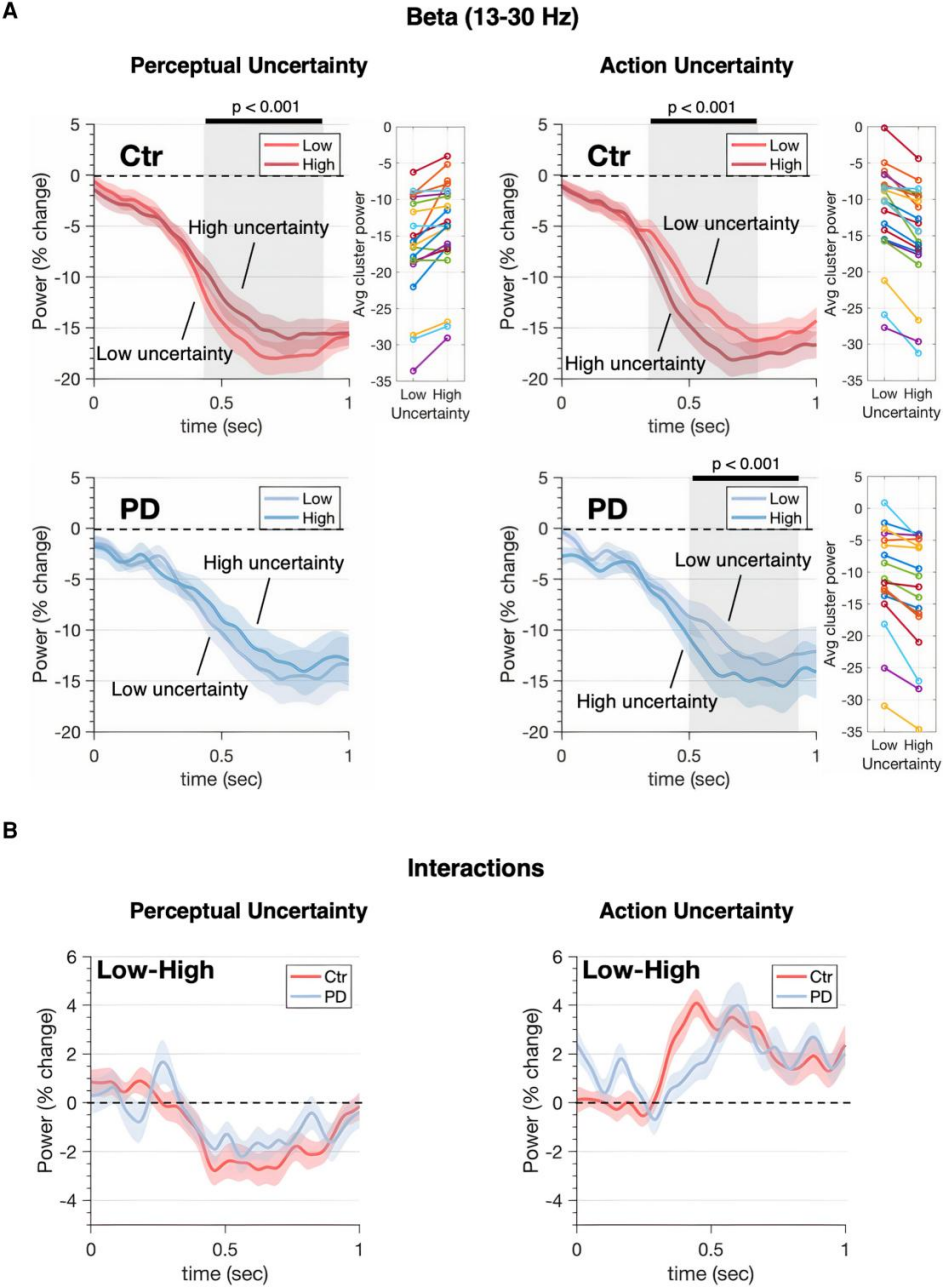
**Uncertainty modulation of beta MEEG oscillatory activity.** Power-envelopes estimated for beta band averaged across trials and ROIs (EEG and MEG modalities fused). (**A**) *Top* row shows power time-series for healthy controls (Ctr). *Bottom* row shows the equivalent data for Parkinson’s disease patients (PD). The onset of coherent motion was followed by desynchronization in beta power. Desynchronization was stronger for low than high perceptual uncertainty, driven by differences in the strength of motion signals. The pattern is reversed for action manipulations where the expected amount of total evidence accumulated scales with the number of options. The effects are significant (with the exception of perceptual manipulation in Parkinson’s disease) and consistent across subjects: panels in the central column show individual beta power averaged within the significant cluster (individual participants' effect of uncertainty are shown in the adjacent colour-coded plot). (**B**) *Bottom* panels show power changes in response to uncertainty between groups (i.e. group × uncertainty interactions). Shaded areas represent SEM, grey shaded rectangles indicate significant differences in power between low and high uncertainty levels. Significance was assessed using cluster-corrected random permutations (10^3^ permutations, nominal α = 0.05, Bonferroni-corrected for four tests: α = 0.0125; Cohen’s *d* = −1.15 for perception and *d* = 1.88 for action comparisons in the beta range).

**Figure 5:**
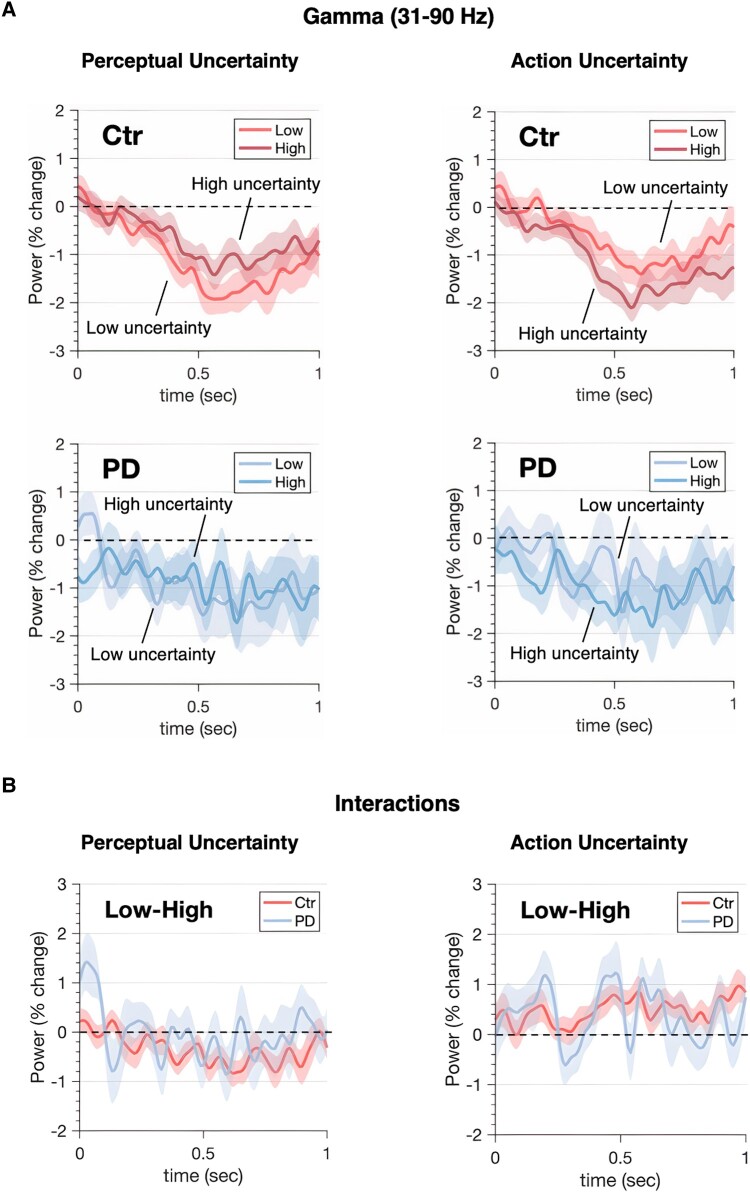
**Uncertainty modulation of gamma MEEG oscillatory activity.** Power-envelopes estimated for both gamma bands averaged across trials and ROIs (EEG and MEG modalities fused). (**A**) *Top* row shows power time-series for healthy controls (Ctr). *Bottom* row shows the same data for Parkinson’s disease patients (PD). Also for gamma the onset of coherent motion was followed by a desynchronization that was stronger for low than high perceptual uncertainty. The pattern is reversed for action manipulations. These effects, however, are not statistically significant. (**B**) *Bottom* panels show power changes in response to uncertainty between groups (i.e. group × uncertainty interactions). Shaded areas represent SEM, grey shaded rectangles indicate significant differences in power between low and high uncertainty levels. Significance was assessed using cluster-corrected random permutations (10^3^ permutations, nominal α = 0.05, Bonferroni-corrected for four tests: α = 0.0125; Cohen’s *d* = −1.15 for perception and *d* = 1.88 for action comparisons in the beta range).

The temporal evolution of the combined MEG and EEG (MEEG) power envelope from each ROI of the parcellated surface served as the signal for our analysis in beta (13–30 Hz) and gamma (31–90 Hz) bands. Confirming results from young healthy adults,^19^ the LBA model predictions were inversely correlated with the MEEG oscillations in beta and gamma bands (Figs 4 and 5).

Specifically, after coherence onset, neural activity desynchronized in a graded fashion and peaked approximately before response suggesting a form of threshold mechanism.^17,52^ For perceptual decisions, the LBA model predicts that the accumulated decision-evidence will ramp quickly with low perceptual uncertainty, and slowly with high perceptual uncertainty.

**Figure 6:**
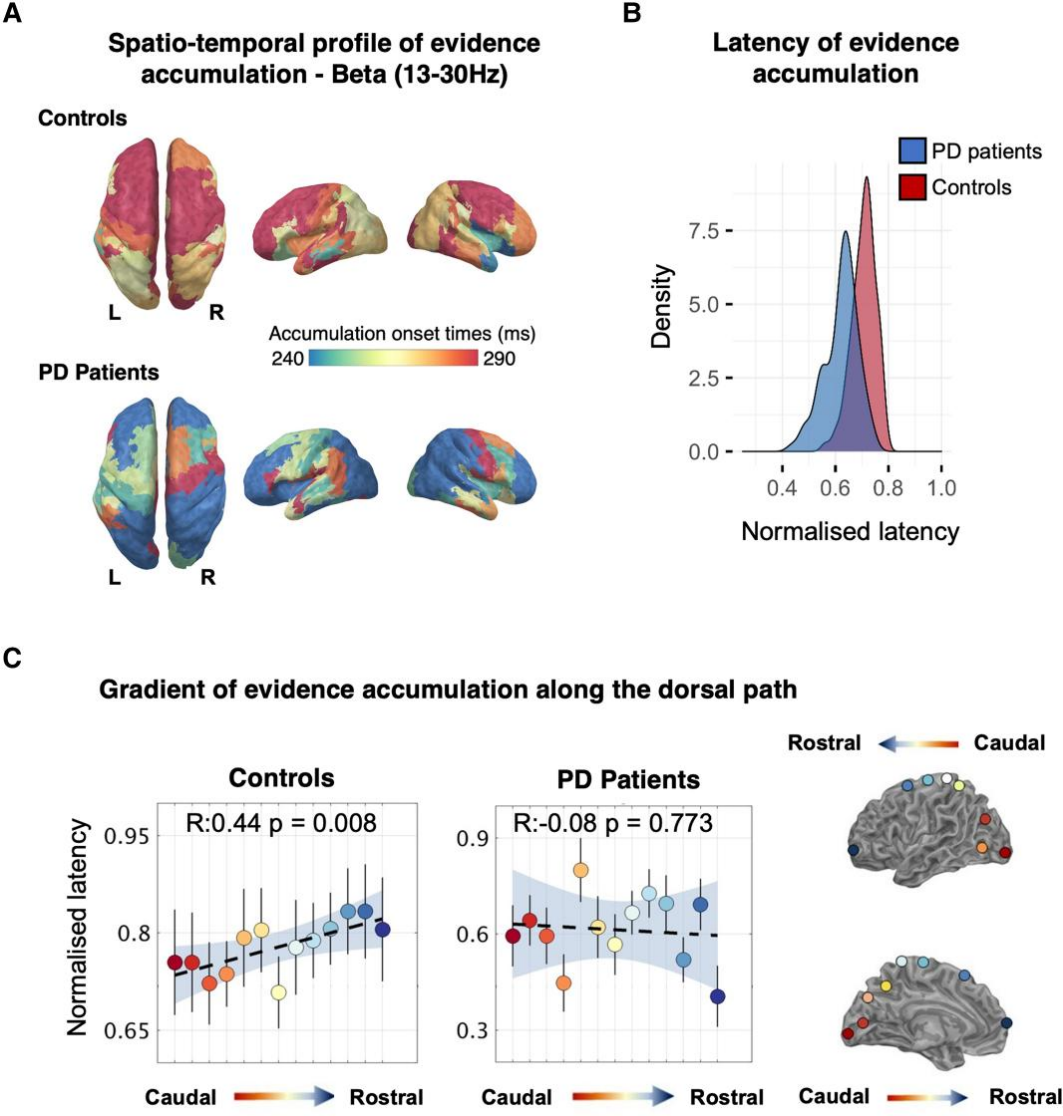
**Temporal cascade of decision-evidence accumulation revealed by comparing trial-by-trial MEEG power-envelopes to model’s predictions.** (**A**) Latency maps showing the normalized latencies (each accumulation onset time divided by individual non-decision time) of decision-evidence accumulation mediated by beta power (13–30 Hz) across anatomical regions where correlations between power-envelopes and model’s predictions survived random permutation testing. A caudo-rostral gradient is visible in the left hemisphere of healthy controls but is disrupted in Parkinson’s disease patients. (**B**) Decision-evidence accumulation in the patient group initiates slightly earlier than in controls. (**C**) Left panel: in healthy controls decision-evidence accumulation mediated by beta follows a caudo-rostral gradient along the dorsal path of the contralateral hemisphere. A linear regression best describes the gradient showing that latencies increase from visual areas up to frontal areas. In the patient group, the caudo-rostral gradient is almost inverted, with frontal regions initiating to accumulate evidence nearly in parallel with visual areas (error bars indicate SEM, shaded area covers bootstrapped 95% regression CI). Right panel: regions of interest (ROIs) along the dorsal path colour-coded with respect to their position along the caudo-rostro axis.

Accordingly, desynchronization of beta power-envelopes averaged across trials and ROIs was larger (*P* = 0.0004 Bonferroni-corrected, cluster-based permutation test) for low than high perceptual uncertainty^19,52^ in controls (Fig. 4). Such a trend was seen in the Parkinson’s disease group (*P* = 0.088 Bonferroni-corrected). When a response is chosen between multiple options, the race underlying the selection of each alternative is characterized by a larger amount of decision-evidence summed across all the racing accumulators by the time of response.^51^ Accordingly, desynchronization of beta power-envelopes averaged across trials and ROIs was larger for high than low action uncertainty in both groups (*P* = 0.0004, Bonferroni-corrected, cluster-based permutation test). Gamma power-envelopes showed a similar trend in the control group, but the effects were statistically insignificant (Fig. 5). Decision-related dynamics expressed by beta desynchronization were distributed across a wide network (Fig. 6A, mean sign-test *Z* across significant ROIs, controls: *Z* = −5.1 ± 0.25, *P* < 0.0001; patients: *Z* = −3.99 ± 0.58, *P* < 0.0001; FDR-corrected) similar to previous reports.^9,19^

Comparisons between *Z*-transformed correlation values from each of the four levels of our manipulations in isolation confirmed that the quality of fit and results did not vary across groups and trial types (*P* > 0.05, FDR-corrected). In the gamma band, we observed a more localized mosaic of ROIs. In the control group, significant ROIs included contralateral motion sensitive areas (inferior lateral occipital region), bilateral extrastriate areas and bilateral frontal regions (comprising frontal pole and superior middle gyrus), ipsilateral motor and supplementary motor area; mean across significant ROIs: sign-test *Z* = −3.22 ± 0.58, *P* = 0.0031, FDR-corrected). In comparison, fewer ROIs survived statistical test in Parkinson’s disease patients (sign-test *Z* = −2.62 ± 0.31, *P* = 0.0084, FDR-corrected) and none of them in the left dorsal path, which is of primary interest for this study. Therefore, from now onwards, the analyses will focus only on beta frequencies.

**Figure 7:**
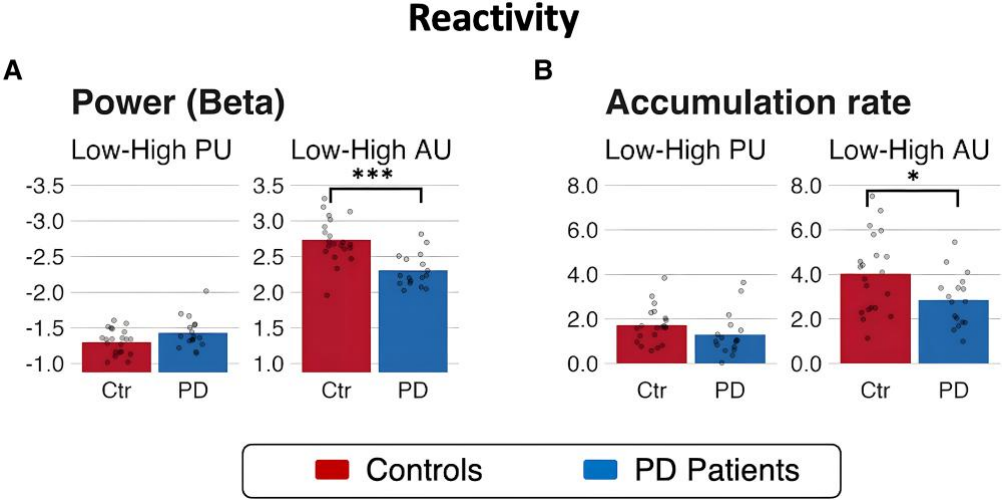
**Parkinson’s disease is associated with reduced flexibility and disrupted gradient of evidence accumulation**. Lower reactivity of beta power (left panel) and accumulation rate (right panel) to varying levels of action uncertainty in the Parkinson’s disease group than in controls. (**A**) A significant interaction between Group and action uncertainty (AU) shows lower reactivity of beta power to varying levels of action uncertainty in the Parkinson’s disease group than in controls (mixed levels effect linear model; *β* = 0.42, CI [0.18, 0.67], *P* < 0.001, *post hoc*: interaction contrast between Low AU–High AU differences between groups: *Z*-ratio = 3.429, *P* = 0.0006, estimate = 0.425, SE = 0.124; Bonferroni-corrected); (**B**) a significant interaction between group and action uncertainty shows lower reactivity of accumulation rates in the patient group than in controls (mixed-design repeated-measures ANOVA: *F*(1,36) = 5.65, MSE = 2.33, *P* = 0.023, ${\hat{\eta }}_{G}^{2}$ = 0.024, BF = 26.95; *post hoc*: interaction contrast between Low AU–High AU differences between groups: *t*(36) = 2.377, *P* = 0.0229, estimate = 1.18, SE = 0.498; Bonferroni-corrected). Significance levels: *P < 0.05, ***P < 0.001.

To explore the moderation effects of disease on beta desynchronization, we fitted a linear mixed effect model to predict power amplitude with Group (controls, patients), PU (low, high) and AU (low, high) as fixed effects, and specified nested subjects and ROIs as random factors. The model’s total explanatory power was substantial (conditional $R^{2}$ = 0.82). The analysis confirmed the effects of perceptual (*β* = 1.30, confidence interval [CI] [1.14, 1.46], *P* < 0.001) and action (*β* = −2.73, CI [−2.90, −2.57], *P* < 0.001) uncertainty. A significant interaction between Group and AU indicated lower reactivity of beta power to varying levels of action uncertainty in the Parkinson’s disease group than in controls (Fig. 7A; *β* = 0.42, CI [0.18, 0.67], *P* < 0.001, *post hoc*: controls High-Low = −2.73, SE = 0.083, *t*-ratio = −33, *P* < 0.0001; patients High-Low = −2.31, SE = 0.092, *t*-ratio = −25, *P* < 0.0001; Bonferroni-corrected). Finally, a non-parametric Wilcoxon rank-sum test confirmed exaggerated beta power (i.e. reduced desynchronization) in Parkinson’s disease compared to controls (dorsal path—both hemispheres: W = 119, *P* = 0.0416, left tailed: controls < patients).

### Evidence accumulation cascade is dysregulated in Parkinson’s disease

To examine the decision-evidence accumulation over space and time, the onset of evidence accumulation across ROIs was identified by optimizing the split of the non-decision time before and after the accumulation period using Spearman correlation to the single-trial MEEG power envelope (Fig. 6A, see ‘Materials and methods’ for details). By tracing the spectrally resolved temporal evolution of decision onset through the visuomotor hierarchy, we found that decision-evidence accumulation emerges with distinct spatiotemporal profiles between healthy controls and Parkinson’s disease patients (Fig. 6A–C). In the control group, we replicated the results from Tomassini *et al*.^19^ Specifically, we show that in the contralateral (i.e. left) hemisphere, the beta-mediated evidence accumulation unfolds in a bottom-up cascade proceeding from caudal sensorial regions to rostral executive areas (Fig. 6A and C).

In people with Parkinson’s disease, accumulation in the beta frequency begins ‘earlier’ than in controls (∼240 ms from coherence onset; Wilcoxon rank-sum: W = 7977, *P* < 0.001). Crucially, the caudo-rostral beta gradient of evidence accumulation is abolished, with frontal regions beginning to accumulate evidence in parallel with caudal visual areas.

The difference between groups is shown in Fig. 6. We fitted a regression model to the mean latencies of ROIs located along the dorsal path for visuomotor decisions^53,54^ (Fig. 6C). For controls (Fig. 6C left top-bottom panels), there is a gradient from caudal to rostral regions (*R*^2^ = 0.44, *P* = 0.008), which contrasts the lack of gradient in Parkinson’s disease patients (*R*^2^ = −0.08, *P* = 0.733). This indicates early decision processes begin in the frontal pole and the middle frontal gyrus, preceding (∼150 ms) onsets in the occipital pole.

## Discussion

The principal result of this study is that Parkinson’s disease alters the spatiotemporal cascade of decisions involved in the transformation from visual stimuli to motor response. Whereas healthy adults manifest a caudal-to-rostral gradient in the latency to onset of evidence accumulation in the beta-frequency range, this gradient is lost in people with Parkinson’s disease (Fig. 6A). Moreover, the accumulation of evidence in the beta range is inflexible in people with Parkinson’s disease, with loss of the modulation according to the perceptual or action uncertainty^2,38,55^ (Fig. 7). Short sensory encoding (i.e. the pre-decision component of the non-decision time) and early accumulation of evidence in the beta range are not confined to classical ‘visual processing’ regions, but are observed throughout the dorsal stream. Indeed, the most striking difference in Fig. 6A is seen over frontal cortical regions, consistent with early proactive rather than later reactive reduction of beta power in frontal sources of top-down influence on the visuomotor decision process. Behaviourally, this was reflected in more errors in Parkinson’s disease (Fig. 3), despite normal cortical volume (Fig. 2).

The use of accumulation-to-threshold modelling enables the identification of anatomical and frequency-specific correlates of the latent cognitive processes underpinning sensorimotor decisions. The modelling of behavioural performance confirmed that increasing levels of perceptual and action uncertainty normally affect decisions by slowing the speed of evidence accumulation in both groups.^2,19,55^ The impact of action uncertainty was different in people with Parkinson’s disease. We refer to the accumulated evidence of action as ‘intention’, to indicate its association with the response rather than stimulus. The accumulation of such action intentions was slower and inflexible in patients compared to controls^2,25^ (Fig. 7A), in keeping with other markers of cognitive inflexibility in Parkinson’s disease.^3,56,57^

In both groups, the within-trial accumulation of evidence was correlated with changes in beta and gamma power. Beta desynchronization has previously been shown to scale with uncertainty.^52^ Here, the temporal profile displays a signature accumulation of decision-evidence over time to a consistent bound that is reached shortly before each movement.^17,58,59^ However, beta desynchronization was impaired by Parkinson’s disease, not only in association with making the response^60-63^ but also in the loss of sensitivity to uncertainty. Note that in our study, cortical beta desynchronization was recorded by MEEG, not the subthalamic beta desynchronization that is also abnormal in Parkinson’s disease.^64,65^ Changes in desynchronization are related to accuracy and latency.^66,67^ Faster beta-mediated flow of decision-evidence through a visuomotor processing hierarchy is associated with faster and more accurate decisions.^19^ During sensory discrimination by non-human primates,^68^ beta desynchronization is greater for accurate trials. Beta desynchronization may represent a general control process not just a determinant of movement. For example, lateralized beta desynchronization correlates with movement preparation, as well as the state of decision-evidence and the updating of a motor plan as decision evolves.^9,17,52,59,69^

In controls, the effect of uncertainty on the evolution of gamma power was qualitatively similar to the effect on beta power, but not significant when permutation corrected for multiple comparisons (Fig. 5), likely due to the lower signal-to-noise ratio of magnetoencephalography at high frequencies. In people with Parkinson’s disease, the effect of uncertainty on gamma power was not seen, but we acknowledge that even the group average data are clearly very noisy. Gamma activity has been proposed to bring neural circuits into a state of readiness for the visuomotor processing,^70^ routing task-relevant information through the integrator units. Such a role may be a specific manifestation of the broader role of gamma oscillations in feed-forward signalling through cortical networks.^71,72^ We attributed the gamma desynchronization to the inhibition of competing motor programs that has been reported for similar tasks requiring alternative actions.^73-75^ In instances like these, the strength of the gamma band diminishes in the sensorimotor cortex as the requirements for movement selection escalate^73,75^ that is consistent with the findings presented here. Nonetheless, the absence of statistical significance for gamma necessitates prudence.

In people with Parkinson’s disease, the change in beta power and loss of beta-reactivity are common signatures of pathology in the basal ganglia and its effect on frontoparietal network function,^23^ and on cognitive or motor processes.^24,76^ Our patients were on their usual dopaminergic medication, which is usually clinically optimized according to motor function, rather than cognition in the absence of clinical cognitive impairment. Deficits in dopamine-dependent pathways may therefore still cause the aberrant generation of oscillatory activity in the beta range during cognitive operations, including decisions for the selection of responses.^23,77^ Our model of evidence accumulation posits that evolving perceptual decisions inform action selection accumulators, enabling a parallel deliberation of available options and motor responses.^8-11^ Posterior regions in health start accumulating evidence first (as soon as sensory information is encoded), but in Parkinson’s disease patients, integration of in occipital and frontal cortex begins near simultaneously. This agrees with previous reports of early visuomotor activation in people with Parkinson’s disease^13^ and suggests that the accumulated evidence (or intention) for action selection is no longer dependent on the evolving perceptual decision, but may instead draw preferentially on prior expectations or perseverated responses.

The normal cascade establishes a compromise between the speed of parallel processes and the accuracy of robust serial decisions. A consequence of the changes in Parkinson’s disease we observe is therefore to improve speed of responding at the potential expense of optimal action selection. The patient responses are correspondingly fast but inaccurate (Fig. 3B and C). Conversely, a delay is typically associated with improved accuracy.^78-81^ Since the aim of sequentially sampling noisy evidence over time is to limit the impact of noise on deliberation, one implication of insufficient sampling before decision onset on downstream accumulators is low quality and less precise (i.e. noisier) information entering the decision process.^58^ The optimal updating of a decision in light of new evidence can also be described as the balanced influence of bottom-up new evidence and top-down prior evidence^9,82^ (Fig. 7B). Noisy sensory information is effectively down-weighted in favour of more precise (stronger) predictions encoded by top-down priors. Recent work on perceptual decision-making^83^ and visuomotor control^84^ suggests shift towards top-down control in people with Parkinson’s disease, which we speculate to arise from the earlier than normal beta power accumulation in prefrontal cortex. Beta oscillations may be a neurophysiological correlate of the estimate of bottom-up uncertainty^84-86^ tracking the trial-by-trial weight of evidence for making decisions,^9^ and the balance between bottom-up evidence versus top-down control during decision-making.^9,84,87-89^

While slower response is typically associated with improved accuracy,^78-81^ patients may be partially compensating for slower action selection and inflexible behaviour by reducing the time allocated to the accumulation of sensory evidence, sacrificing accuracy to keep behavioural reactions within an ecologically balanced range. However, we note that this proposal would imply strategic control over the information processing cascade that might be lacking in Parkinson’s disease, as suggested by the reduced sensitivity of beta desynchronization to uncertainty. Moreover, a recent study^90^ that sought to investigate this very proposition was unable to arrive at a definitive conclusion. This highlights the need for further investigation into the balance between bottom-up evidence and top-down control during decision-making, as well as the potential compensatory behaviours employed by individuals with Parkinson’s disease.

There are limitations to our study. We rely on a clinical diagnosis, without evidence of Lewy-body neuropathology in our patient cohort. Moreover, we report the time of symptom onset to scan, rather than diagnosis to scan, because of uncertainty over the timing at which the individuals’ diagnosis were made with grounds for clinical confidence. Our sample size was modest, although in keeping with medium to large effect sizes in previous work on motor control and action selection in Parkinson’s disease, and large in the context of the task-based magnetoencephalography literature. Further, our participants were on their usual medication and we cannot confirm the dopaminergic basis of the effects we observe, as opposed to other anatomical and neurochemical consequences of the disease. Nonetheless, we did confirm that our participants did not have dementia or marked cognitive impairment, or significant cortical atrophy. Our ask is complex and required training to reach the standardized performance thresholds. It was possible that patients might not be able to learn the task, and would have had to be excluded, although this did not arise for our cohort of people with mild Parkinson’s disease. The MEEG method does not detect subcortical signals, but is restricted to cortical neurophysiology. The lack of atrophy, together with the condition-specific and frequency-specific effects we observed, makes it unlikely that a non-specific cognitive impairment is the cause of the observed abnormalities. Nonetheless, we are agnostic as to whether the observed neurophysiological changes are a direct result of cortical pathology of indirect consequence of subcortical degeneration in cortico-striato-thalamo-cortical circuits and their dopaminergic innervation.

In conclusion, we have demonstrated the integration of cortical physiological recordings with LBA models of decisions, based on sensory evidence and motor intentions. The normal cascade of temporally overlapping decisions, with a rostro-causal gradient of latency of beta-mediated accumulation, is absent in Parkinson’s disease. This is accompanied by insensitivity of the beta power to uncertainty, representing the failure to modify decision processes in the face of uncertainty that is ordinarily required to optimize behavioural decisions.

## Data Availability

Raw data were generated at the MRC Cognition and Brain Sciences Unit. Derived data and code to reproduce the findings of this study are available at https://github.com/ale-tom/MEG_LBA_PD.
